# Recurrence of Lemierre’s Syndrome Presenting With Extensive Cerebral Venous Sinus Thrombosis: A Case Report

**DOI:** 10.7759/cureus.95452

**Published:** 2025-10-26

**Authors:** Soe Latt Tun, Thi Han, Aye Thinzar Moe, Ingyin May

**Affiliations:** 1 General Medicine, King's College Hospital NHS Foundation Trust, London, GBR

**Keywords:** case report, cerebral venous sinus thrombosis (cvst), jugular vein thrombosis, leimerre', recurrent thrombosis, septic thrombophlebitis

## Abstract

Lemierre’s syndrome is a rare but potentially life-threatening condition characterised by septic thrombophlebitis of the internal jugular vein following oropharyngeal infection, most commonly caused by *Fusobacterium necrophorum*. Although it typically affects young, otherwise healthy adults, recurrent or chronic presentations in older individuals are uncommon and diagnostically challenging. We report a 43-year-old male with a prior episode of Lemierre’s syndrome secondary to a peritonsillar abscess complicated by left internal jugular vein thrombosis and septic pulmonary emboli, who presented with severe left-sided headache, neck pain, vomiting, and transient visual disturbances following recent antibiotic treatment for presumed tonsillitis. Imaging revealed extensive cerebral venous sinus thrombosis involving the superior sagittal, transverse, and sigmoid sinuses, without evidence of active oropharyngeal infection or septic focus. The patient had discontinued long-term anticoagulation six weeks before admission because of medication unavailability and a possible drug-related rash. He was treated with therapeutic-dose enoxaparin during admission and was subsequently transitioned back to rivaroxaban upon discharge, with follow-up imaging demonstrating partial recanalisation of the affected sinuses. This case highlights the potential for recurrent or chronic thrombotic complications of Lemierre’s syndrome in the absence of overt infection and underscores the importance of sustained anticoagulation, early recognition of cerebral venous sinus involvement, and multidisciplinary follow-up to prevent late complications in high-risk patients.

## Introduction

Lemierre’s syndrome is a rare but potentially life-threatening condition, first described by André Lemierre in 1936, characterised by septic thrombophlebitis of the internal jugular vein (IJV) following an oropharyngeal infection [1]. The condition most commonly arises as a complication of tonsillitis, pharyngitis, or peritonsillar abscess and is classically associated with *Fusobacterium necrophorum*, an anaerobic Gram-negative bacillus, although other organisms such as *Streptococcus *species have also been implicated [1,2]. Despite the widespread use of antibiotics, the syndrome continues to be reported, with an estimated incidence of approximately 3.6 cases per million annually, highlighting its clinical relevance even in the modern antibiotic era [2].

The pathophysiology typically involves local invasion of the lateral pharyngeal or parapharyngeal space, leading to inflammation and subsequent thrombosis of the IJV. This can result in septic embolisation, most frequently to the lungs, but may also involve other sites such as the joints, liver, or central nervous system [3,4]. Cerebral venous sinus thrombosis (CVST) is an uncommon but serious complication that can occur due to extension of the IJV thrombus into the dural venous sinuses [3,5].

Although Lemierre’s syndrome predominantly affects previously healthy young adults, atypical presentations in older adults, outside the usual demographic range, and recurrent or chronic cases have been reported [6,7]. Such presentations may occur in the context of underlying prothrombotic disorders, immunocompromise, or interrupted anticoagulation therapy [1,7]. Recurrence or persistence of thrombosis despite clinical improvement raises diagnostic and therapeutic challenges and underscores the need for vigilant long-term follow-up.

We present the case of a 43-year-old man with a previous history of Lemierre’s syndrome who re-presented with extensive CVST in the setting of interrupted anticoagulation and polycythaemia. This case highlights the potential for late or recurrent thrombotic complications, even in the absence of active oropharyngeal infection, and emphasises the importance of sustained anticoagulation and multidisciplinary management in such patients.

## Case presentation

A 43-year-old man with a history of chronic smoking and previous alcohol excess presented with a three-week history of left-sided neck pain, initially described as a dull ache with occasional sharp, shooting episodes. The pain progressed into a worsening left-sided headache, which became excruciating over the two days before admission. This was associated with episodes of vomiting and transient blurred vision in the left eye, particularly exacerbated by forward bending. Two weeks earlier, he had experienced odynophagia, which was treated by his general practitioner with oral antibiotics for presumed tonsillitis. Although swallowing improved, the headache persisted.

**Figure 1:**
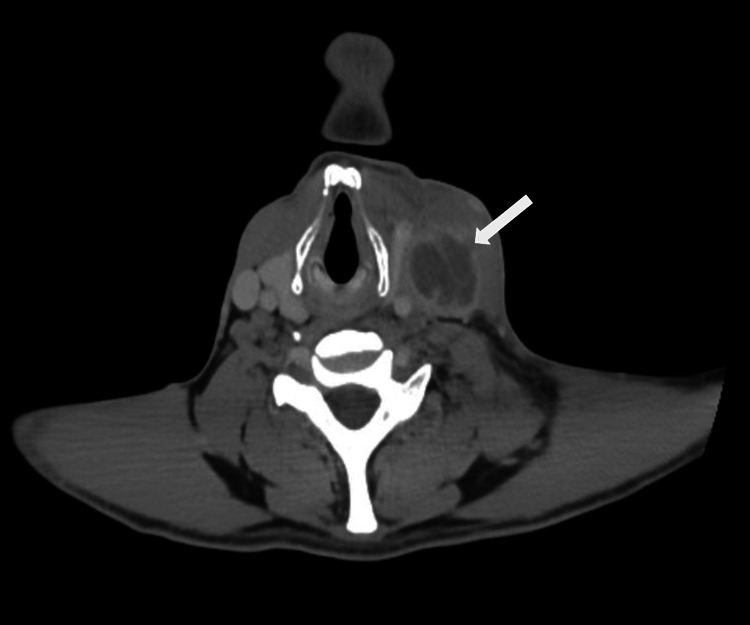
Contrast-enhanced CT of the neck from a previous admission showing left internal jugular vein thrombosis (white arrow) at the level of the upper cervical region related to initial episode of Lemierre's syndrome

His past medical history included an unprovoked pulmonary embolism, Lemierre’s syndrome secondary to a peritonsillar abscess complicated by left IJV thrombosis and septic pulmonary emboli (Figure 1), and secondary polycythaemia. He had been advised to remain on lifelong anticoagulation but had discontinued rivaroxaban six weeks before presentation because of medication unavailability. He also reported intolerance to apixaban, which had been associated with a persistent, non-pruritic rash of uncertain aetiology. His ability to attend follow-up was limited by poor digital access, and he had missed multiple outpatient appointments.

He lived with his father, and his partner resided nearby. He used a basic keypad phone and relied on postal letters for communication and appointment reminders, as he did not have access to a smartphone or email. He had no learning difficulties or cognitive impairment but reported difficulty keeping track of appointments. He smoked approximately 20 cigarettes per day, occasionally used cannabis, and had a history of previous alcohol excess but reported minimal current intake.

On examination, he was haemodynamically stable. Neurological assessment was unremarkable, with no cranial nerve deficits or signs of meningism. Laboratory investigations revealed neutrophilic leucocytosis (WBC 16.1 × 10⁹/L), persistent polycythaemia (Hb 201 g/L), and a mildly elevated C-reactive protein (9 mg/L) (Table 1). These findings suggested an inflammatory response without evidence of active sepsis, consistent with a predominantly prothrombotic rather than infective process. Given the absence of current oropharyngeal symptoms or a clear infectious source, antibiotics were not initiated. Blood cultures were not obtained as the patient was afebrile, had no clinical features of sepsis, and inflammatory markers were only mildly elevated.

**Table 1:** Laboratory investigations on admission MCV: mean corpuscular volume, eGFR: estimated glomerular filtration rate, CKD-EPI: chronic kidney disease epidemiology collaboration, ALP: alkaline phosphatase, CRP: C-reactive protein.

Parameters	Reference	Results
White cell count	2.9-9.6 10 × 9/L	16.1
Haemoglobin	125-170 g/L	201
Haematocrit	0.390-0.510 L/L	0.578
MCV	81-100 fL	108
Platelet count	140-400 10 × 9/L	252
Neutrophils	1.50-6.10 10 × 9/L	13.47
Lymphocytes	0.80-3.50 10 × 9/L	1.25
Sodium	135-145 mmol/L	134
Potassium	3.5-5.3 mmol/L	4.6
Urea	2.5-7.8 mmol/L	1.6
Creatinine	61-123 µmol/L	58
eGFR by CKD-EPI (2009)	NA mL/min/1.73 m^2^	>90
Alkaline phosphatase (ALP)	30-130 U/L	145
Total bilirubin	<21 µmol/L	23
CRP	<5 mg/L	9

**Figure 2:**
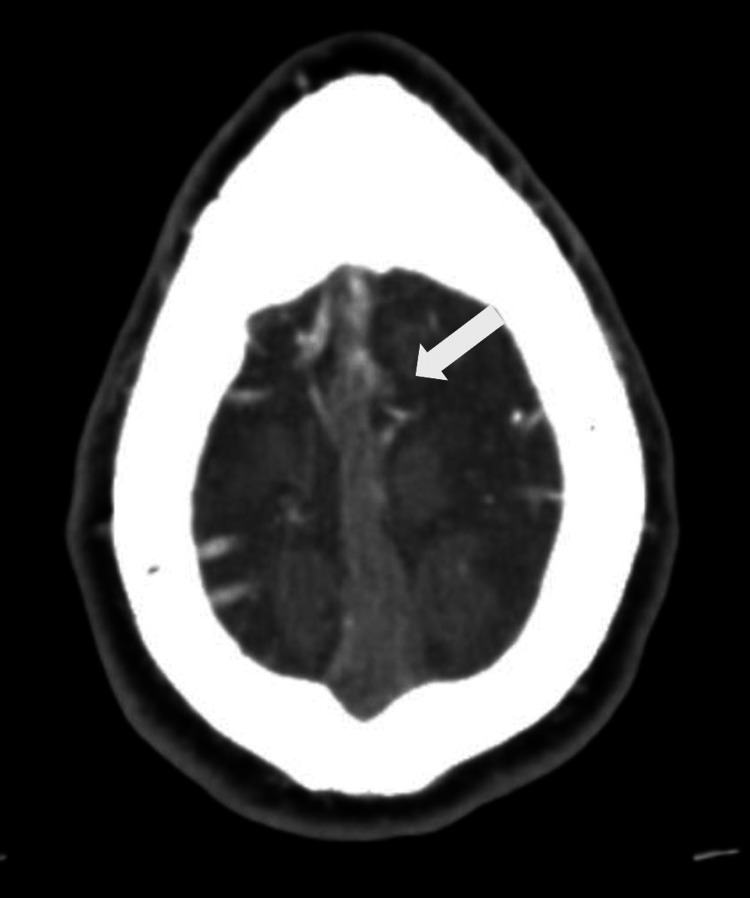
CT venogram demonstrating thrombosis of superior sagittal sinus (white arrow)

**Figure 3:**
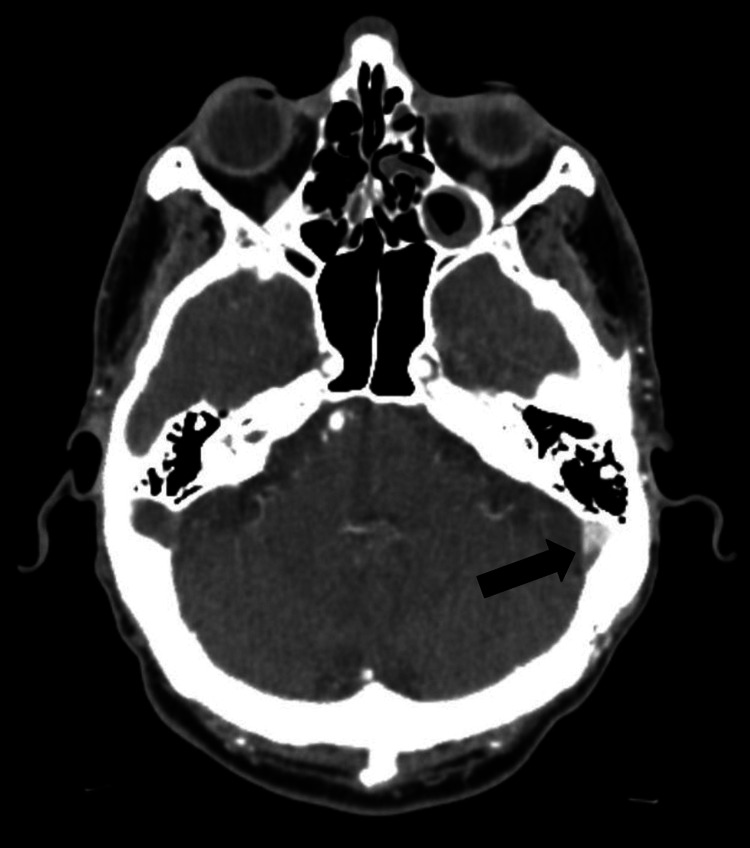
CT venogram showing left transverse sinus thrombosis (black arrow)

**Figure 4:**
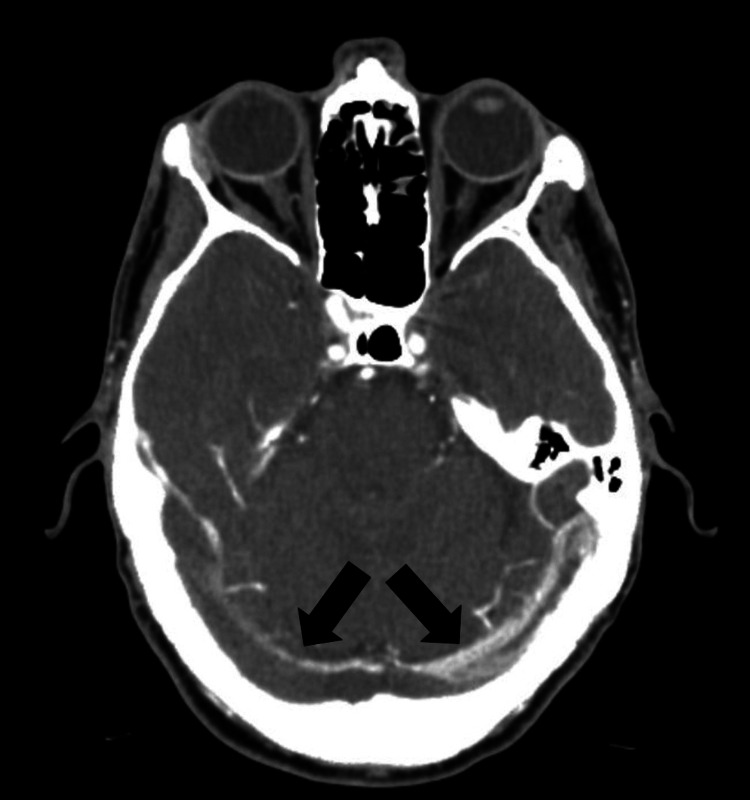
CT venogram demonstrating bilateral sigmoid sinus thrombosis (black arrows), with filling defects in both sigmoid sinuses, more pronounced on the right

CT venography demonstrated extensive CVST involving the superior sagittal sinus (Figure 2), confluence of sinuses, left transverse sinus (Figure 3), and bilateral sigmoid sinuses (Figure 4). He was commenced on therapeutic enoxaparin and later transitioned back to rivaroxaban. Following a nine-day hospital admission, he was discharged with outpatient anticoagulation follow-up. A repeat CT venogram five weeks after discharge showed partial recanalisation of the superior sagittal, left transverse, and left sigmoid sinuses, with residual filling defects in the right transverse and sigmoid sinuses suggestive of chronic venous sinus thrombosis. Clinically, his symptoms improved with resolution of headache and visual blurring. Secondary polycythaemia persisted and is now being managed with a venesection plan under haematology supervision, aiming to maintain haematocrit below 0.45.

Compliance with anticoagulation was reinforced before discharge, and follow-up was arranged via telephone rather than mailed letters to accommodate the patient’s communication limitations. Ongoing review was organised through the anticoagulation clinic and his general practitioner to ensure prescription continuity and medication supply. Smoking cessation advice was provided, and haematology follow-up was arranged for continued monitoring of secondary polycythaemia and long-term anticoagulation management.

Haematology review following discharge confirmed secondary polycythaemia with normal erythropoietin levels. Bone marrow aspirate was hypocellular with normal morphology, and trephine biopsy demonstrated trilineage haematopoiesis without excess blasts, consistent with a reactive rather than myeloproliferative process.

A summary of the patient’s clinical course, from the initial episode of Lemierre’s syndrome to the current recurrence, is presented in Table 2.

**Table 2:** Summary of clinical timeline

Timeframe	Clinical Events/Findings	Management/Outcome
Year 1	Unprovoked Pulmonary embolism diagnosed	Commenced on apixaban, later switched to rivaroxaban due to rash
Year 0 (First Leimerre’s episode)	Peritonsillar abscess complicated by left internal jugular vein thrombosis and septic pulmonary emboli (Leimerre’s syndrome)	Treated with intravenous antibiotics and anticoagulation. Recommended life long anticoagulation
6 weeks prior to recurrence	Patient discontinued rivaroxaban after running out of tablets and missing follow-up appointments	No anticoagulation
Current admission	Presented with severe, progressive left-sided headache and neck pain. No oropharyngeal symptoms or sepsis	Initial CT showed non-opacification of right sigmoid sinus. CT venogram confirmed extensive cerebral venous sinus thrombosis. Commenced on therapeutic enoxaparin
Discharge and follow-up	Transitioned to rivaroxaban on discharge after 9 days of admission	Follow up CT venogram after 5 weeks showed partial recanalisation of affected sinues

## Discussion

This case highlights the critical importance of ongoing anticoagulation in patients with a history of Lemierre’s syndrome, particularly when presenting with oropharyngeal symptoms [1]. Although Lemierre’s syndrome is classically described in otherwise healthy adolescents and young adults, this case illustrates a rare recurrence and chronic sequela in an older individual outside the typical demographic range, with additional prothrombotic risk factors [2,3]. The patient had previously experienced Lemierre’s syndrome with left IJV thrombosis and septic pulmonary emboli due to *Streptococcus anginosus* (Group C), which was managed with antibiotics and anticoagulation [2,3]. His recent interruption of lifelong anticoagulation, together with contributory factors such as secondary polycythaemia and tobacco use, likely predisposed him to thrombus propagation and recurrence [1]. There are currently no standardised international guidelines for the management of Lemierre’s syndrome; treatment strategies are largely based on case reports and expert consensus, emphasising prolonged broad-spectrum antibiotic therapy and individualised use of anticoagulation for extensive thrombosis or embolic complications [1,3,4,7].

Lemierre’s syndrome typically follows oropharyngeal infections, most often pharyngitis or peritonsillar abscess, but it may also arise from other deep neck space infections, including otitis media, mastoiditis, and dental or parapharyngeal infections [2,7]. In many cases, the initial oropharyngeal symptoms have resolved by the time IJV thrombosis and systemic signs of infection become evident [5]. Progression into septic thrombophlebitis of the IJV usually occurs within one to three weeks of symptom onset and may be accompanied by spiking fevers and tenderness along the sternocleidomastoid muscle [5]. However, in some patients, particularly those with posterior compartment involvement of the parapharyngeal space, local signs may be subtle or absent [7].

In this case, the absence of overt oropharyngeal signs on admission did not exclude the possibility of septic progression [5,6]. The patient’s social vulnerabilities, including limited access to digital communication, missed follow-up appointments, and poor medication adherence, further complicated disease management. This underscores the importance of multidisciplinary involvement, including infectious disease, haematology, and primary care, to ensure long-term monitoring, education, and recurrence prevention in high-risk individuals [1]. These social barriers, including reliance on postal letters and difficulty maintaining regular follow-up, directly contributed to treatment interruption and likely played a key role in the recurrence of thrombosis in this patient.

The decision to withhold antibiotics was guided by the absence of clinical or biochemical evidence of active infection, supported by mild CRP elevation and lack of fever or oropharyngeal findings. Therapeutic anticoagulation was prioritised given the extensive cerebral venous involvement and the patient’s known prothrombotic risk factors, in line with published case-based management approaches [1,3,4]. The patient’s secondary polycythaemia and elevated haemoglobin further supported a hypercoagulable state, contributing to thrombus propagation despite normal platelet counts and only modest inflammatory activity.

Recurrence was considered related to the previous Lemierre’s syndrome, given the involvement of the same venous drainage pathway as the prior IJV thrombosis, in the absence of a new infective focus or alternative provoking factor. Although imaging did not demonstrate residual chronic thrombus, the patient’s persistent prothrombotic risk factors, including secondary polycythaemia and recent interruption of lifelong anticoagulation, support the interpretation of a recurrent or propagated thrombotic process within a previously affected venous territory rather than a new thrombotic event.

Recurrent or delayed thrombotic sequelae after Lemierre’s syndrome are biologically plausible due to ongoing endothelial injury within the internal jugular-dural venous system, residual intimal changes, and a sustained proinflammatory or hypercoagulable state following the initial septic thrombophlebitis [1]. Recurrence or progression can occur particularly when patient-level thrombotic risks persist (for example, polycythaemia and smoking) or when anticoagulation is interrupted, even in the absence of a new oropharyngeal source [2,3,7]. CVST is thought to arise through retrograde extension from the internal jugular bulb into the sigmoid and transverse sinuses [3,5].

Evidence suggests that most patients recover fully with timely initiation of antibiotics and anticoagulation; however, chronic venous occlusion, pulmonary sequelae, or intracranial thrombosis may still develop when thrombus burden is extensive or risk factors persist [1,2,7]. Anticoagulation remains individualised and is primarily supported by case-based evidence. Many authors advocate for therapeutic anticoagulation in cases involving extensive IJV thrombosis, cerebral venous sinus extension, or septic emboli, typically initiating treatment with low-molecular-weight heparin followed by an oral agent [3,4]. Reported treatment durations vary widely from six weeks to six months, with longer courses often recommended for patients with ongoing prothrombotic conditions or recurrent disease [1,3,4,7].

Our management, which involved initial treatment with enoxaparin followed by rivaroxaban and interval imaging demonstrating partial recanalisation, aligns with current case-based practices, although standardised anticoagulation protocols remain undefined. Long-term outcomes are most favourable when anticoagulation adherence is maintained and modifiable risk factors, such as smoking and haematological abnormalities, are concurrently addressed through coordinated multidisciplinary follow-up [1,7].

Secondary polycythaemia and chronic smoking likely contributed to this patient’s hypercoagulable state. Both conditions are associated with increased blood viscosity, endothelial dysfunction, and enhanced platelet activation, all of which predispose to venous thrombosis. Chronic hypoxia from smoking may further stimulate erythropoiesis, compounding the prothrombotic risk. These factors, combined with interrupted anticoagulation, may have acted synergistically to trigger recurrent venous sinus thrombosis despite the absence of active infection [1,3,7].

This case contributes to the existing literature by demonstrating that recurrent or chronic thrombosis can occur in patients with a previous episode of Lemierre’s syndrome even in the absence of active infection, highlighting that endothelial injury and persistent prothrombotic risk factors may play an ongoing role. Unlike most reported cases, this recurrence occurred several months after apparent clinical recovery, emphasising the need for sustained anticoagulation adherence and multidisciplinary follow-up. The case also illustrates the impact of social determinants, such as limited digital access and inconsistent follow-up, on clinical outcomes, an aspect seldom discussed in prior reports.

## Conclusions

This case illustrates the importance of maintaining a high index of suspicion for septic thrombophlebitis in patients with a history of Lemierre’s syndrome, even in the absence of current oropharyngeal symptoms or an identifiable septic focus. The development of extensive CVST in this patient, despite initial resolution of infective symptoms, demonstrates that septic complications may recur or persist beyond the acute phase, particularly in those with underlying prothrombotic risk factors and inconsistent treatment adherence. It also reinforces the critical role of long-term anticoagulation in preventing thrombotic progression in patients with prior Lemierre’s syndrome. Furthermore, this case highlights how social determinants of health, such as digital exclusion and barriers to medication access, can significantly affect follow-up and outcomes. A multidisciplinary and patient-centred approach is essential to ensure continuity of care, adherence to lifelong anticoagulation, and timely recognition of late or recurrent complications.
